# The Cerebellar Response to Visual Portion Size Cues Is Associated with the Portion Size Effect in Children

**DOI:** 10.3390/nu16050738

**Published:** 2024-03-05

**Authors:** Bari A. Fuchs, Alaina L. Pearce, Barbara J. Rolls, Stephen J. Wilson, Emma J. Rose, Charles F. Geier, Hugh Garavan, Kathleen L. Keller

**Affiliations:** 1 Department of Nutritional Sciences, The Pennsylvania State University, University Park, PA 16802, USA; baf44@psu.edu (B.A.F.); azp271@psu.edu (A.L.P.); bjr4@psu.edu (B.J.R.); 2 Department of Psychology, The Pennsylvania State University, University Park, PA 16802, USA; sjw42@psu.edu (S.J.W.); ejr5384@psu.edu (E.J.R.); 3 Human Development and Family Science, University of Georgia, Athens, GA 31793, USA; charles.geier@uga.edu; 4 Department of Psychological Sciences, University of Vermont, Burlington, VT 05405, USA; hgaravan@uvm.edu; 5 Department of Food Science, The Pennsylvania State University, University Park, PA 16802, USA

**Keywords:** fMRI, portion size, food cue reactivity, eating behavior

## Abstract

The neural mechanisms underlying susceptibility to eating more in response to large portions (i.e., the portion size effect) remain unclear. Thus, the present study examined how neural responses to portion size relate to changes in weight and energy consumed as portions increase. Associations were examined across brain regions traditionally implicated in appetite control (i.e., an appetitive network) as well as the cerebellum, which has recently been implicated in appetite-related processes. Children without obesity (i.e., BMI-for-age-and-sex percentile < 90; N = 63; 55% female) viewed images of larger and smaller portions of food during fMRI and, in separate sessions, ate four meals that varied in portion size. Individual-level linear and quadratic associations between intake (kcal, grams) and portion size (i.e., portion size slopes) were estimated. The response to portion size in cerebellar lobules IV–VI was associated with the quadratic portion size slope estimated from gram intake; a greater response to images depicting smaller compared to larger portions was associated with steeper increases in intake with increasing portion sizes. Within the appetitive network, neural responses were not associated with portion size slopes. A decreased cerebellar response to larger amounts of food may increase children’s susceptibility to overeating when excessively large portions are served.

## 1. Introduction

Serving larger portions of food increases intake across a variety of food types [1] and environmental settings [2,3]. The tendency to consume more food in terms of weight and energy when exposed to larger portions (i.e., “the portion size effect”—PSE) emerges as early as infancy [4] and is evident across the lifespan [1]. The PSE is persistent up to five days with no compensation evident in children, leading to sustained increases in energy intake [5]. As excess energy intake is associated with the development of obesity and long-term health risks [6,7], it is important to understand the neurobiological mechanisms that underly this behavior.

The control of eating behavior involves a network of cortical (e.g., prefrontal cortex, insula) and subcortical (e.g., basal ganglia, amygdala, hippocampus, hypothalamus) regions that integrate exteroceptive signals (e.g., visual portion size cues) with interoceptive signals from the gut and periphery [8,9]. This network contributes to a range of processes relevant for appetitive behavior, including energy homeostasis, perception, reward responsiveness, learning, memory, cognitive regulation, and interoception [9,10,11]. The presentation of visual food cues (e.g., images) elicits patterns of neural engagement across the appetitive network [12] that are associated with eating behaviors in both children [13,14] and adults [15,16,17]. For example, prior analyses from our group [14] found that greater activation to images of larger (vs. smaller) portions of food in the orbitofrontal cortex (OFC) and ventromedial prefrontal cortex (vmPFC) was positively associated with children’s food intake in response to larger food portions, whereas the opposite relationship was observed in the inferior frontal gyrus (IFG). These data suggest that susceptibility to the PSE is associated with neural responses to food portion size cues in brain regions implicated in valuation (e.g., OFC, vmPFC) and cognitive control (e.g., IFG). Nevertheless, these analyses only examined neural responses in a limited number (n = 9) of regions using a region-of-interest-based approach; thus, a more comprehensive voxel-wise examination of the appetitive network is needed to identify regions sensitive to the PSE.

Although the cerebellum is typically excluded or minimally integrated into models of eating behavior [8,9,10,11], it is involved in many processes that may contribute to food intake including sensorimotor [18], reward [19,20], affective [21], and cognitive [22,23] processing. Growing evidence suggests that the cerebellum contributes to hunger and satiety processing [24,25], meal anticipation [26,27], taste processing [28], visual food cue processing [29,30,31,32], and satiation/meal cessation [33] through connections with cortical and subcortical regions [25,33]. For example, cerebellar neurons respond to food cues and nutrients, and their activation can suppress food intake via signals to subcortical reward pathways [34]. In humans, there is evidence that cerebellar responses to food cues (e.g., images, commercials) differ by the adolescent weight status [30], child-reported loss of control eating [35], and parent-reported child food responsiveness [29], suggesting that cerebellar processing of visual food cues may contribute to pediatric eating behaviors. Therefore, it is important to characterize how cerebellar processing of food cues relates to objectively measured intake in youth.

Our group’s prior examination of associations between neural food cue reactivity and the PSE tested a cohort that was heterogeneous in body weight [14]. However, since the weight status is associated with both neural responses to food cues [36] and the PSE [5], including children with and without obesity may confound results. Taking this into account, the present study aimed to examine how neural responses to food images varying in portion size relate to the PSE in children without obesity. To do so, children who had BMI-for-age-and-sex percentiles < 90 viewed images of larger and smaller portions of food during functional magnetic resonance imaging (fMRI), and individual-level PSEs were estimated from intake of four laboratory meals that varied in portion size. We hypothesized that children’s patterns of intake across the four portion size meals would relate to their neural responses to portion size within an a priori-defined appetitive network and the cerebellum. More specifically, we anticipated that increased susceptibility to the PSE would be associated with greater activation to larger vs. smaller portion size cues in regions implicated in reward and value processing (e.g., OFC, vmPFC) and less engagement in regions implicated in cognitive control (e.g., dorsolateral PFC, IFG), interoception (e.g., insula), and satiation (e.g., cerebellum).

## 2. Materials and Methods

### 2.1. Participants

As part of a 7-visit, prospective study aiming to identify risk factors for pre-adolescent obesity (ClinicalTrials.gov, NCT03341247), 88 (45 male, 43 female) children (mean [SD] age = 7.8 [0.62] years) attended 6 baseline visits. Children were accompanied by the parent primarily responsible for feeding decisions and were required to be 7–8 years old and have a BMI-for-age-and-sex percentile < 90 (i.e., not have obesity). Children were ineligible if they were colorblind, not reading at grade level, not fluent in English, had a learning (e.g., dyslexia) or neurodevelopmental disorder (e.g., ADHD), had a diagnosed psychological condition (e.g., anxiety), were taking medications known to influence appetite, cognition, or blood flow, or had any MRI contraindications (e.g., metal in the body, claustrophobic). Consent and assent were obtained in accordance with the Institutional Review Board of The Pennsylvania State University.

Neural responses to food and non-food cues in this cohort has been examined in a separate paper [37], and the PSE has been examined in a larger behavioral sample [38]. The associations between food cue reactivity and the PSE in the present publication have not been reported elsewhere.

### 2.2. Data Collection

**Figure 1:**
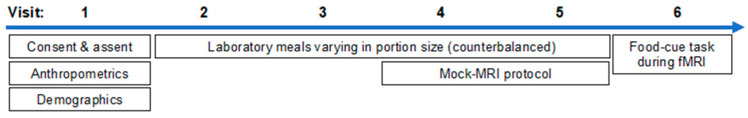
Timeline of procedures related to the present analyses. During visit 1, parental consent and child assent were obtained in accordance with the Institutional Review Board of The Pennsylvania State University, anthropometrics were assessed, and parents reported demographic information. During visits 2–5, children were served four meals that varied in portion size. During visits 4 and 5, children completed a 2-session mock-MRI protocol to familiarize them with the scanning environment. During visit 6, children completed a food-cue task during functional magnetic resonance imaging (fMRI).

Data for the present analyses were collected across six baseline visits between 2017 and 2022, with an ~8-month interruption due to the COVID-19 pandemic (study protocol and materials are available on Open Science Foundation at https://osf.io/ynjqw/, accessed on 21 February 2024). Procedures pertinent to the present analyses include the collection of demographics (see Section 2.2.1) and anthropometrics (see Section 2.2.2), assessment of parent-reported child appetitive traits (see Section 2.2.3), laboratory meals to assess the PSE (see Section 2.2.4), a mock-MRI protocol (see Section 2.2.5), and a food-cue task during fMRI (see Section 2.2.6 and Section 2.2.7; Figure 1). Parents were instructed to have their child fast for at least 3 h prior to each visit to create a physiological state similar to what would be experienced prior to a meal.

#### 2.2.1. Demographics

Parents completed a questionnaire assessing child race and ethnicity, yearly family income, and parental education (see Table 1). Family income and maternal education were included as separate indicators of the socioeconomic status [39].

#### 2.2.2. Anthropometrics

Children’s height and weight were measured twice by a trained researcher using a standard scale (Scale Tronix model 5002, Welch Allyn, Chicago, IL, USA; precision to nearest 0.1 kg) and stadiometer (precision to nearest 0.1 cm), respectively. Children removed shoes and heavy clothing items prior to measurement. The BMI-for-age-and-sex percentile was computed using averaged height and weight measurements based on growth charts from the Centers for Disease Control and Prevention [40].

#### 2.2.3. Laboratory Portion Size Meals

Across four visits, children were served four laboratory meals that varied in the amount of food served [38]. Meals contained varied amounts of macaroni and cheese, chicken nuggets, broccoli with margarine, and grapes, as well as fixed amounts of ketchup, and ad libitum access to water. The smallest portion size condition was the reference amount, and subsequent conditions increased all food weights by 33%, 66%, and 99% relative to the reference amount (Table S1). Reference amounts were based on the Continuing Survey of Food Intake by Individuals [41] and previous laboratory meal paradigms [14,42]. The order of portion sizes was randomly assigned and counterbalanced across the four meals. Foods were weighed before and after meals to the nearest 0.1 g, and nutrition facts labels or reliable online databases (https://fdc.nal.usda.gov/, accessed on 20 December 2023) were used to convert the weight consumed to kcal.

Prior to each meal, children reported liking in response to tasting small samples (~2–3 g) of the meal foods using a five-point facial hedonic scale. They also reported fullness using a child-friendly visual analogue scale before and after each meal [43]. Children were told they had 30 min to eat ad libitum until they reached satiation. If they reached satiation before 30 min, they could notify the researcher. Children were notified during the meal when 15 min had passed and when they had a few minutes remaining. Meals were served in an observation room that had a table and chairs, a rug with child-friendly images (e.g., trains), and non-food pictures (e.g., animals) on the wall. To serve as a neutral distraction during meals, children were read an age-appropriate, non-food-related book by either a trained researcher or computer (i.e., audio book); methodologies were consistent per child but switched during the COVID-19 pandemic to comply with social distancing restrictions. Mealtimes (i.e., lunch or dinner) varied across children based on participant availability but were consistent per child.

#### 2.2.4. Mock-MRI Protocol

Children completed a two-session mock-MRI protocol [43] to familiarize them with the scanning environment. During the first session, children visited the mock scanning room and had an opportunity to ask questions. During the second session, children completed a mock scanning protocol where they entered a mock MRI bore, viewed non-food images, experienced simulated MRI sounds, and practiced using the response grip to make ratings.

#### 2.2.5. fMRI Visit Protocol

Children arrived for the fMRI visit following at least a 3 h fast. As individuals with and without an overweight status show an increased food cue response during fasted conditions [44], we scanned children in the neutral appetitive state. To achieve this, children who reported fullness levels below 25% using a child-friendly visual analogue scale [45] upon arrival were given a snack (6.75 fl oz of apple juice, Quaker Chewy granola bar) and then re-rated their fullness after consumption. This process was repeated a second time if a child continued to report fullness below 25%. The fullness rating collected closest to the scan was used as the index of pre-MRI fullness (imputed for one participant due to recording error; see Supplement Materials). Before and after the scan, children rated their state anxiety using the Children’s Anxiety Meter Scale [46].

#### 2.2.6. MRI Data Acquisition

A 3 Tesla MRI scanner (Magnetom Trio model, Siemens, Malvern, PA, USA) and 20-channel head coil were used to collect imaging data. A T1-weighted MPRAGE (160 sagittal slices; TR = 1650; TE  =  2.03 ms; flip angle  =  9°; FOV  =  256 × 256 mm^2^; voxel size = 1 × 1 × 1 mm^3^) was acquired using generalized autocalibrating partially parallel acquisition (GRAPPA; acceleration factor 2) [47]. During each run of the food-cue task (~2.7 min), 80 T2*-weighted images were acquired using echo planar imaging (EPI; slice thickness = 3 mm (no gap); 33 descending slices; TR  =  2000 ms; TE  =  26 ms; flip angle  =  90°; FOV  =  220 × 220 mm^2^; voxel size = 3 × 3 × 3 mm^3^). To optimize the temporal lobe and cerebellar signal based on prior findings relevant to our aims [29], EPI scans were aligned parallel to the AC–PC line and adjusted vertically. Each EPI scan was preceded by two unsaved “dummy scans” to establish a stable magnetization state prior to data collection. Lastly, a field map was acquired using a double-echo gradient-echo sequence (slice thickness = 3 mm (no gap), 33 descending slices, TR  =  400 ms, TE 1  =  5.12 ms, TE 2 = 7.65 ms, flip angle  =  60°, FOV  =  220 × 220 mm^2^, voxel size = 3 × 3 × 3 mm^3^).

#### 2.2.7. Food-Cue Task

**Figure 2:**
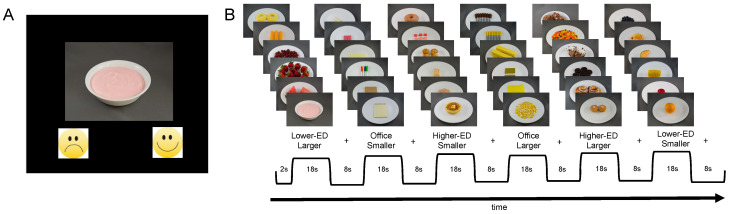
Food-cue task. Children viewed images of food and non-food (i.e., office supply) items from a standardized dataset [47] in a block design. Images were categorized into six conditions with 30 images each: (1) larger amount of higher-energy-dense (ED) food, (2) larger amount of lower-ED food, (3) smaller amount of higher-ED food, (4) smaller amount of lower-ED food, (5) larger amount of office supplies, and (6) smaller amount of office supplies. Images were presented over five ~2.7 min runs. (**A**) Image presentation: Images were presented on a black screen above a smiley face and a frowny face. For each image, children answered the question “do you want this?” by pressing one of two buttons with their dominant hand (index finger for “no”/frowny face and thumb for “yes”/smiley face). (**B**) Example run: Each run contained one block per image condition. Block orders were pseudorandomized across runs. Each block contained six images. Each image was presented for 2.0 s followed by a 0.5 s fixation cross on a black screen. Each block was followed by an 8.0 s fixation cross on a black screen.

During the food-cue task, children viewed images of 120 food items and 60 non-food items (i.e., office supplies) presented one at a time (Figure 2). Images were sourced from a standardized dataset [47] and presented above smiley and frowny faces on a black screen (Figure 2A). To encourage engagement and attention during the task, children were instructed to indicate whether they wanted each item by pressing one of two buttons with their dominant hand (index finger for “no”/frowny face and thumb for “yes”/smiley face). Food images were categorized based on the amount (i.e., portion size; larger, smaller) and energy density (ED; higher: <1.5 kcal/g, lower: <1.5 kcal/g), creating four conditions with 30 images each: (1) a larger amount of higher-ED food, (2) larger amount of lower-ED food, (3) smaller amount of higher-ED food, and (4) smaller amount of lower-ED food. Food items depicted included those served during the meals (macaroni and cheese, chicken nuggets, broccoli, and grapes) as well as other foods commonly consumed by children (e.g., strawberries, cake, deli meat, pizza). Portions depicted in the smaller-amount condition were based on servings from nutrition facts labels and reflected amounts consumed per eating occasion by children in the 1990s [41]. Office supplies were also categorized based on the amount (larger, smaller), creating two conditions. Images were presented in a block design over five runs; each run began with a 4.0 s fixation and contained one block (n = 6 images) per cue condition (i.e., six blocks per run; Figure 2B). Each image was presented for 2.0 s followed by a 0.5 s fixation. Each block was followed by an 8.0 s fixation. Children viewed blocks in one of two pseudorandomized and counterbalanced orders.

### 2.3. fMRI Data Processing

Standard preprocessing steps were implemented using fMRIPrep 20.2.3 [48] (see Supplementary Materials for full report). MNI’s unbiased template for pediatric cohort 3 (MNIped; ages 7–11 y) [49,50] was used for volume-based spatial normalization. Brain masks from BOLD signal (i.e., BOLD brain masks) and head-motion parameters were estimated from functional scans. Susceptibility distortion correction was applied to functional scans using a field-map-based approach (n = 62) or field-map-less approach (n = 1) if field-map quality was poor. Functional scans were also slice-time corrected (i.e., realigned) to the middle of each TR, co-registered to anatomical space, and resampled into MNIped space. Preprocessed data were visually inspected using summary reports generated by fMRIPrep. If quality of preprocessed data was poor, scans were excluded (see Section 2.4.1) or reprocessed. Data were subsequently blurred with a 6 mm FWHM Gaussian kernel, scaled so results could be represented as percent signal change, and analyzed (see Section 2.4.5) using AFNI v21.3.04 [51,52].

Individual-level general linear models (GLMs) included 1 parameter per food cue condition and were modeled by convolving block onset times and durations with the canonical hemodynamic response function. Onset times were shifted by subtracting half a TR (i.e., 1 s) to account for fMRIPrep’s default slice-time realignment to the middle of each TR. In addition, GLMs included 14 nuisance regressors computed by fMRIPrep (6 rigid-body motion parameters and their derivatives, average signal within CSF and white matter masks). To ensure analyzed data were acquired during a stable magnetization state, the first two volumes of each run and any additional steady-state outliers identified by fMRIPrep were censored. To reduce the effects of motion, volumes with a framewise displacement [53] > 0.9 mm were censored, matching the motion threshold used by the Adolescent Brain Cognitive Development (ABCD^®^) Study [54]. Runs with >20% of volumes censored across task blocks were excluded from GLMs. A first-level portion size contrast (larger–smaller food amounts, across ED) was generated from each GLM.

### 2.3.1. Masks

**Figure 3:**
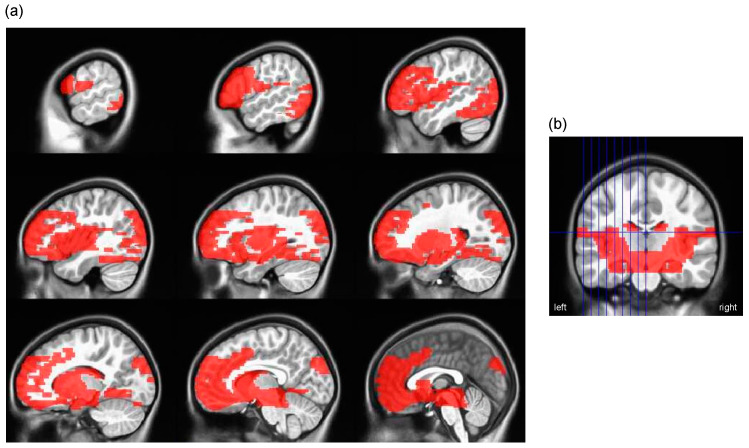
Appetitive network mask: (**a**) Sagittal slices with appetitive network mask (red) overlayed on MNI’s unbiased template for pediatric cohort 3. Regions included in mask are listed in Table 1; (**b**) coronal slice with appetitive network mask (red) overlayed on MNI’s unbiased template for pediatric cohort 3. Blue vertical lines indicate location of sagittal slices depicted in (**a**).

Separate appetitive network and cerebellum masks were generated using the Wake Forest University (WFU) PickAtlas toolbox for Statistical Parametric Mapping 12 (SPM12 [55]). Masks were dilated by 4 mm to avoid small, isolated regions and irregular boundaries and restricted to voxels scanned in at least 80% of children (e.g., to only include voxels inside the brain). Regions in the appetitive network mask (Table 1) were selected based on previous reviews [11,56,57]; cortical regions were defined using Brodmann areas; and subcortical regions were defined based on anatomical boundaries (Figure 3). The cerebellum was anatomically defined (Figure S1).

### 2.4. Group-Level Analyses

#### 2.4.1. Analysis Sample

Children were excluded from analyses if they declined to enter the MRI (n = 4), did not complete at least 3 runs of the food-cue task (n = 4), or had fewer than 3 runs included in first-level analyses (see Section 2.3; n = 17). Children were also excluded from some analyses of eating behavior if they had data for <4 meals (due to measurement error) and thus could not be included in quadratic fixed-effects individual slopes models (n = 3; see Section 2.4.2). In addition, children were excluded from analyses within the appetitive mask if they had a reduced cortical field of view identified via visual inspection (n = 2). In sum, sample sizes for analyses ranged from N = 58 to 63.

#### 2.4.2. Estimation of Individual-Level Portion Size Slopes

The PSE (i.e., relationship between portion size and intake) was estimated for both weight (g) and energy (kcal) of food consumed. As large portions of food have been shown to increase intake in a curvilinear manner [58,59], we tested both linear and quadratic PSE models: (1) intake (g or kcal)~intercept + linear portion size, and (2) intake (g or kcal)~intercept + linear portion size + quadratic portion size. In subsequent sections, we refer to the linear slope of the linear model as the “linear portion size slope” and the quadratic slope of the quadratic model as the “quadratic portion size slope”. Meal portion size was modeled using the proportion increase from the reference portion (i.e., 0, 0.33, 0.66, 0.99) so that a 1-unit increase in portion size reflects the estimated change in intake between the reference portion and approximately the largest portion (100% increase). Fixed-effects individual slope (FEIS) models were used to extract child-specific slope estimates for the linear and quadratic PSE models (feisr 1.3.0 [60] in R 4.2.2 [61]). Both models were adjusted for meal-related influences on intake (i.e., pre-meal fullness, average pre-meal liking of foods at the meal, and meal order).

#### 2.4.3. Descriptive Statistics

Histograms and Kolmogorov–Smirnov tests were used to assess the normality of linear and quadratic portion size slopes and imaging covariates (i.e., pre-MRI fullness, pre-MRI anxiety, framewise displacement). The mean and standard deviation are reported for normally distributed quantitative variables while quartiles (25% (Q1), 50%, 75% (Q3)) are reported for non-normally distributed variables. Pearson correlations were used to assess associations between linear and quadratic portion size slopes estimated for weight and energy consumed (Table S2). Associations between portion size slopes and child characteristics (age, BMI percentile, sex) were assessed using Pearson correlations for age and BMI percentile and 2-sample t-tests for sex (Table S3). Demographic variables and imaging covariates were compared between children included and excluded from analyses using 2-sample *t*-tests and *χ*^2^ or Fisher’s Exact Test for continuous and categorical variables, respectively.

#### 2.4.4. Wanting Responses during the Food-Cue Task

Using lme4 1.1-32 [62] in R 4.2.2 [61], a mixed-effects linear model with the subject as the random effect was used to compare the percent of items children reported wanting per block (%want) during the food-cue task between larger and smaller portion size conditions; models predicted %want from portion size (larger, smaller), controlling for the run. The estimated marginal means (EMMs) and corresponding 95% confidence intervals from this model are reported for larger and smaller portion size conditions.

To explore whether wanting responses might mediate the observed association between neural responses and the PSE, a post hoc correlation was conducted between the average difference in %want between larger and smaller portion size conditions and the neural response to portion size (larger–smaller) extracted from the cerebellar cluster associated with the quadratic portion size slope identified in fMRI analyses (see 2.4.5).

#### 2.4.5. Neural Responses to Portion Size and the PSE

Within each mask (see Section 2.3.1), four regression analyses (3dttest++) were used to test associations between the neural response to portion size (larger–smaller) and child-specific portion size slopes; models included parameter estimates from (a) linear or (b) quadratic PSE models (see Section 2.4.2) estimated from (a) weight (g) or (b) energy (kcal) of food consumed. The predictors of interest include the linear and quadratic portion size slopes. Since individual slope estimates (e.g., linear slope) cannot be interpreted outside the context of the overall curve for each child (e.g., linear slopes depend on intercepts), all child-specific FEIS model coefficients were included in regression models (i.e., linear model: intercept + linear slope; quadratic model: intercept + linear slope + quadratic slope). Models were adjusted for average framewise displacement, sex, pre-MRI fullness, and pre-MRI anxiety to control for their potential effects on the BOLD response. Within each mask, cluster thresholds derived from 3dClustStim [63] were used to adjust for multiple comparisons (voxel-wise threshold *p* < 0.001, cluster-corrected to *p* < 0.05). To examine whether significant results were confounded by the weight status, sensitivity analyses were conducted by including the BMI percentile as a covariate.

## 3. Results

### 3.1. Participant Characteristics

The analyzed sample was predominantly White (n = 61; 97%) and non-Hispanic/Latinx (n = 63; 100%), had family incomes > USD 50,000 and had mothers with a bachelor’s degree or higher (Table 2). Children included and excluded from analyses did not differ in demographics (*ps* > 0.59), pre-MRI anxiety (*p* > 0.05), or pre-MRI fullness (*p* > 0.88). As expected, children included in analyses exhibited less motion (i.e., average framewise displacement) during the food-cue task than those excluded (t(19) = −4.6, *p* < 0.001).

### 3.2. Child-Specific Portion Size Curves

Child-specific linear portion size slopes (adjusted for meal order, average liking, and fullness) ranged from a decrease of 194 g or 205 kcal to an increase of 370 g or 647 kcal between the reference and largest portion sizes (Table 3). Despite the large range, 76% and 73% of children had positive linear portion size slope estimates for weight and energy consumed, respectively, indicating that most children increased intake with increasing portions as expected. Adjusting for meal order, average liking, and fullness, 38% (n = 23) of children had positive quadratic portion size slopes estimated from weight consumed, while 50% (n = 37) had positive quadratic portion size slopes estimated from energy consumed. Example quadratic portion size curves can be found in Figure S2.

### 3.3. Behavioral Responses during the Food-Cue Task

The median response rate during the food-cue task was 97.5% (Q1 = 95.8%, Q3 = 99.2%). The percentage of items wanted per block (%want) did not significantly differ between larger (EMM = 66.9; 95% CI: 62.1–71.6) and smaller (EMM = 68.3; 95% CI: 63.6–73.1) portion size conditions (F(1, 1141.2) = 1.02, *p* = 0.31). The average difference in %want between larger and smaller portion size conditions was not significantly correlated with the neural response to portion size (larger–smaller) in the cerebellar cluster associated with the quadratic portion size slope (see Section 3.4.2; r(56) = −0.50, *p* = 0.62).

### 3.4. Associations between Neural Responses to Portion Size and the PSE

#### 3.4.1. Appetitive Network Mask

In models adjusted for framewise displacement, sex, pre-MRI fullness, pre-MRI anxiety, and FEIS model coefficients, there was no association between the linear or quadratic portion size slopes (weight or energy) and neural response to portion size (larger vs. smaller) within the appetitive network (*p* > 0.05).

#### 3.4.2. Cerebellum Mask

**Figure 4:**
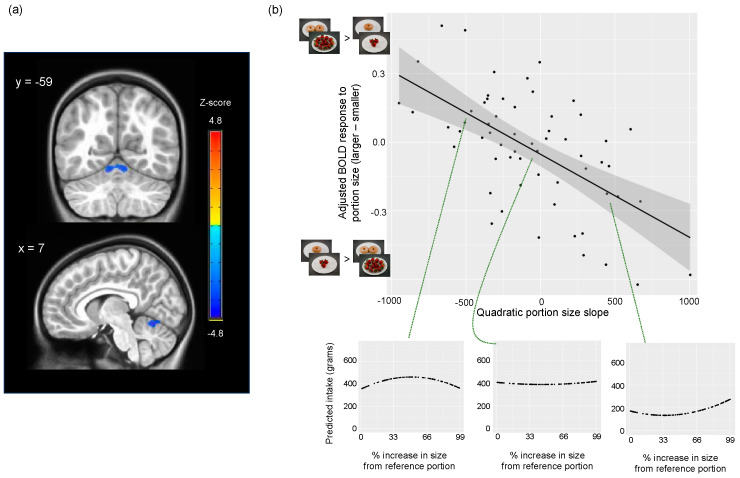
Association between cerebellar response to portion size (larger–smaller) and quadratic portion size slope estimated from gram intake (N = 58): (**a**) z-statistic map for prediction of BOLD response to portion size (larger–smaller) by quadratic portion size slope controlling for sex, average framewise displacement, pre-MRI fullness, and pre-MRI anxiety, and intercept and linear slope from quadratic FEIS model. Analyses were conducted within cerebellum mask. Quadratic portion size slope was negatively associated with activation in cluster (blue) spanning cerebellum lobules IV-VI (peak: 7, −62, −13; k = 1853 1 × 1 × 1 mm^3^ voxels; peak z-statistic = −4.83); (**b**) scatterplot of adjusted BOLD response to portion size (larger–smaller; *y*-axis) vs. quadratic portion size slope (*x*-axis). BOLD responses to portion sizes were extracted from cluster depicted in (**a**) and adjusted for covariates included in imaging analyses. Using individual-level intercepts and slopes estimated from quadratic FEIS models, portion size curves (i.e., predicted gram intake vs. percent increase in portion size from reference portion) were plotted for three subjects (circled in green) to exemplify patterns of intake for children with negative (left), approximately zero (middle), and positive (right) quadratic slopes.

Adjusting for the same variables listed above (Section 3.4.1), the quadratic portion size slope estimated from gram intake was negatively associated with the neural response to portion size (larger–smaller) in cerebellar lobules IV–VI (MNI peak: 7, −62, −13; cluster size = 1853 1 × 1 × 1 mm^3^ voxels; peak z-statistic = −4.83; Figure 4 and Figure S3). More positive quadratic portion size slopes were associated with lower activation to images depicting larger compared to smaller food portions while more negative quadratic portion size slopes were associated with greater activation to images depicting larger compared to smaller food portions. Therefore, children who showed steeper increases in weight consumed with increasing portion sizes had lower cerebellar activation to images depicting larger compared to smaller food portions. In contrast, children who showed plateaus of or steeper declines in weight consumed with increasing portion sizes had greater cerebellar activation to images depicting larger compared to smaller food portions (see Figure 4). Results were similar after additionally adjusting for the BMI percentile (MNI peak: 7, −61, −13; cluster size = 1935 1 × 1 × 1 mm^3^ voxels; peak z-statistic = −4.94). Cerebellar responses to portion size were not associated with linear portion size slopes (g or kcal) or the quadratic portion size slope estimated from kcal.

## 4. Discussion

The present study examined how neural responses to food portion size relate to the PSE in children without obesity. Results revealed an association between food cue activation in the cerebellum (culmen, declive) and the amount (weight) of food consumed across meals varying in portion size. As cerebellar activation to larger (relative to smaller) portion sizes increased, children exhibited plateaus or steeper declines in intake with increasing portion sizes. Conversely, as cerebellar activation to larger (relative to smaller) portion sizes decreased, children exhibited steeper increases in intake with increasing portion sizes. These findings suggest that decreased cerebellar activation in response to larger amounts of food may increase children’s susceptibility to overeating when faced with “supersized” portions of food.

Cerebellum lobules IV–VI (culmen, declive) are implicated in multiple processes relevant to feeding behavior [34], including leptin sensitivity [24], chewing [64], food cue reactivity [29,30], reward anticipation and expectation [19], and emotional conditioning [65]. For example, in adults with leptin deficiency, a greater number of days of receiving leptin replacement was associated with greater lobule VI activation to high-energy food vs. non-food images [24], suggesting hormones that influence satiety alter food cue responding in the cerebellum. The function and structure of lobules IV–VI have also been associated with self-reported eating behavior (e.g., disinhibition [66], loss of control [35,67]) and weight status [24,68,69,70]. These findings, along with our observation that activation in lobules IV–VI relates to objectively measured food intake in children, indicate that cerebellar processes contribute to susceptibility to overconsumption.

With the addition of the present findings, there is growing evidence that intact cerebellar function supports the development of satiation [25,71] and meal cessation [33]. For example, in mice, the activation of anterior deep cerebellar nuclei reduced meal size and feeding duration, leading to reductions in food intake [33]. This change was mediated by blunted phasic dopamine responses to food consumption, suggesting the cerebellar induction of satiation via reward-related pathways (i.e., hedonic satiation [34]). Low and colleagues [33] proposed that the cerebellum fine-tunes predictive reward signals by integrating information about the expected nutrient value from food (or food cues) with the physiological nutrient status. Over the course of a meal, cerebellar neurons sense the nutrient status and induce dopamine efflux, leading to an eventual blunting of food-dependent phasic dopamine responses that is necessary to reduce the value of further consumption and bring about meal cessation [33]. Thus, children who have reduced cerebellar activation to larger portions of food may not be adequately fine-tuning predictive reward signals based on the nutrient status during a meal, enabling sustained motivation to eat and delayed meal cessation when they are presented with excessively large portions. This postulation aligns with behavioral research demonstrating that increased susceptibility to the PSE in children is associated with decreased sensitivity to internal fullness signals (i.e., satiety responsiveness) and increased sensitivity to external food cues (i.e., food responsiveness) [5]. Alternatively, in line with behavioral research showing associations between the PSE and executive functions [38], the cerebellum may influence satiation via the modulation of cognitive processes [72,73]. PFC–cerebellar interactions are thought to enable the optimization of behaviors according to context [74], and the modulation of PFC–cerebellar interactions has been shown to modulate food-induced appetite and hunger in individuals with obesity [25]. As the cerebellum is implicated in a variety of additional processes that could underlie its association with child appetitive behavior (e.g., oral behaviors [75,76], ocular control to visualize objects of interest [77], affect [78]), our interpretations are speculative, and additional research is needed to delineate the role of the cerebellum in meal cessation and the PSE.

In contrast to our hypotheses, we did not observe associations between portion size slopes estimated from weight or energy consumed and neural responses within the appetitive network (i.e., a mask of cortical and subcortical regions implicated in the control of food intake). This is in contrast to a previous study in 7–10-year-old children [14] where linear and quadratic relationships between portion size and intake were modulated by neural responses to portion size cues in the OFC, vmPFC, IFG, and caudate. Differences in results may be due to demographic differences between the samples: the present sample was younger (7–8 years) than the sample in [14] and did not include children with obesity. As puberty influences the development of brain regions implicated in reward and cognitive control [79], associations observed in older children may not generalize to younger children. Further, neural responses to portion size in brain regions associated with valuation and cognitive control may not influence food intake prior to the development of excess adipose tissue. In addition, differences in results may be due to methodology: the present study examined susceptibility to the PSE with fixed-effect individual slope models rather than mixed-effects models and examined voxel-wise associations within a masked area rather than using a region-of-interest-based approach. The two studies also used different data processing methodology (e.g., choice of structural atlas, use of spatial smoothing) and software, which could impact results. To better understand how neural responses within the appetitive network relate to the PSE, future research should test the moderating effects of puberty and the weight status and compare analytic methods.

This study has several strengths. First, we objectively measured food intake to quantify intake rather than using self-reported measures of dietary behavior, which are subject to misreporting [80]. Second, intake was assessed at four laboratory meals, allowing us to capture both linear and quadratic relationships between intake and portion size. Third, using a voxel-wise analytic approach within masks allowed us to explore associations between individual-level portion size slopes and neural responses within a larger brain area than previous region-of-interest-based analyses [14]. Despite these strengths, the sample size of the present study was smaller than proposed (https://www.clinicaltrials.gov/study/NCT03341247, accessed on 20 December 2023) due to data loss (e.g., from motion) and difficulty recruiting during the COVID-19 pandemic. Small sample sizes can increase the risk of both false negatives and false positives [81]; thus, results will need to be replicated in other samples. In addition, the sample was predominately White and not Hispanic, so results may not generalize to more diverse populations. Further, while objective assessment of food intake mitigates error compared to self-report methods, eating behaviors observed in the laboratory may not generalize outside the laboratory environment. Lastly, the identified association between cerebellar function and intake was observational and does not indicate causality. The causal relationship between cerebellar function and the portion size effect could be tested empirically by modulating cerebellar activation (e.g., with transcranial direct current stimulation, as performed in [25]) prior to assessments of food intake.

This is the first study to examine how neural responses to portion size cues across the appetitive network and cerebellum relate to changes in objectively measured intake as portion sizes increase. The present results suggest that decreased cerebellar activation to large portion size cues in children without obesity may be a risk factor for overeating in response to large portions. Based on prior studies [25,33,71], reduced activation may influence the PSE by increasing hunger or reducing satiation via reward or cognitive mechanisms; nevertheless, additional research is needed to test this. If future research supports a causal mechanism between cerebellar function and the PSE, interventions that modulate cerebellum activity (e.g., non-invasive stimulation [82], mindfulness training [83], cognitive training [84], cognitive behavioral therapy [85]) may mitigate increased intake in response to large portions.

## Figures and Tables

**Table 1:** Regions in appetitive network mask.

Region	Brodmann Area (s)
Dorsolateral prefrontal cortex	9, 46
Anterior prefrontal cortex	10
Orbitofrontal cortex	11, 12
Insula	13, 14, 16
Associative visual cortex	19
Subgenual area	25
Dorsal anterior cingulate cortex	32
Fusiform	37
Primary gustatory cortex	43
Inferior frontal gyrus	44, 45, 47
Hippocampus	-
Hypothalamus	-
Globus pallidus (lateral, medial)	-
Caudate (body, tail, head)	-
Putamen	-
Substantia nigra	-
Subthalamic nucleus	-
Amygdala	-
Midbrain	-

**Table 2:** Participant characteristics by analysis inclusion status.

	Included (N = 63)	Excluded (N = 25)
**Sex**		
Male	30 (47.6%)	15 (60.0%)
Female	33 (52.4%)	10 (40.0%)
**Age, yrs**		
Mean (SD)	7.82 (0.587)	7.79 (0.595)
Min, Max	7.00, 8.99	7.03, 8.81
**Family Income**		
<USD 51,000	9 (14.3%)	2 (8.0%)
USD 51,000–USD 100,000	28 (44.4%)	14 (56.0%)
>USD 100,000	24 (38.1%)	8 (32.0%)
Missing	2 (3.2%)	1 (4.0%)
**Maternal Education**		
<Bachelor’s Degree	13 (20.6%)	5 (20.0%)
Bachelor’s Degree	30 (47.6%)	11 (44.0%)
>Bachelor’s Degree	19 (30.2%)	9 (36.0%)
Missing	1 (1.6%)	0 (0%)
**BMI Percentile**		
Mean (SD)	47.0 (25.5)	49.0 (24.2)
Min, Max	3.91, 89.3	6.69, 89.5
**Pre-MRI Fullness**		
Mean (SD)	71.3 (30.1)	70.2 (33.2)
Min, Max	4.00, 150	3.00, 139
Missing	1 (1.6%)	0 (0%)
**Pre-MRI Anxiety**		
Median [Q1, Q3]	2.0 [0.0, 3.0]	3.0 [0.75, 6.0]
Min, Max	0, 10	0, 10
Missing	0 (0%)	1 (4.0%)
**Average Framewise Displacement**		
Median [Q1, Q3]	0.30 [0.19, 0.38]	1.52 [0.89, 1.67]
Min, Max	0.0800, 1.29	0.500, 4.79
Missing	0 (0%)	6 (24.0%)

**Table 3:** Descriptive statistics for portion size slopes estimated from weight (g) and energy (kcal) consumed.

Portion Size Slope	N	Mean (SD)	Min, Max
Linear portion size slope, g ^a^	63	58.0 (113)	−194, 370
Linear portion size slope, kcal ^b^	63	124 (175)	−205, 647
Quadratic portion size slope, g ^c^	60	−66.8 (419)	−953, 1090
Quadratic portion size slope, kcal ^d^	60	−26.8 (628)	−1500, 1470

^a^ Slope extracted from linear fixed-effects individual slope (FEIS) model predicting weight consumed (g). ^b^ Slope extracted from linear FEIS model predicting energy consumed (kcal). ^c^ Slope extracted from quadratic FEIS model predicting weight consumed (g). ^d^ Slope extracted from quadratic FEIS model predicting energy consumed (kcal).

## Data Availability

Data and analytic code described in the manuscript are available on Open Science Framework (https://osf.io/cnwk4/, accessed on 21 February 2024) and OpenNeuro (https://openneuro.org/datasets/ds004697/versions/1.0.2, accessed on 20 December 2023).

## Supplementary Materials

The following supporting information can be downloaded at: https://www.mdpi.com/article/10.3390/nu16050738/s1, Figure S1: Cerebellum mask; Table S1: Amount of food served by portion size condition; Table S2: Pearson correlation coefficients between individual-level portion size slopes; Table S3: Associations between portion size slopes and child characteristics; Figure S2: Example portion size curves for gram intake; Figure S3: Association between cerebellar response to portion size and quadratic portion size slope with example portion size curves. References [37,86,87] are cited in Supplementary Materials.
