# Supplementary material for: The Cerebellar Response to Visual Portion Size Cues Is Associated with the Portion Size Effect in Children
Source: Nutrients. 2024 Mar 5;16(5):738. doi: 10.3390/nu16050738 (PMC10933954; doi:10.3390/nu16050738)
Supplement: Supplementary file 1 [file nutrients-16-00738-s001.zip › nutrients-2810163-supplementary.pdf]

Cerebellar response to visual portion size cues is associated with the portion size effect in children

Bari A. Fuchs, Alaina L. Pearce, Barbara J. Rolls, Stephen J. Wilson, Emma J. Rose, Charles F. Geier, Hugh Garavan, and Kathleen L. Keller

Affiliations:

Department of Nutritional Sciences, Pennsylvania State University (BAF, ALP, BJR, KLK)

Department of Psychology, Pennsylvania State University, University Park, PA, USA (SJW, EJR)

Human Development and Family Science, University of Georgia, Athens, GA USA (CFG)

Department of Psychological Sciences, University of Vermont, Burlington VT, USA (HG)

Department of Food Science, Pennsylvania State University, University Park PA, USA (KLK)

Supplementary Data

Methods

fMRI pre-processing

Results included in this manuscript come from preprocessing performed using fMRIPrep 20.2.3 (Esteban, Markiewicz, et al. (2018); Esteban, Blair, et al. (2018); RRID:SCR_016216), which is based on Nipype 1.6.1 (Gorgolewski et al. (2011); Gorgolewski et al. (2018); RRID:SCR_002502).

Anatomical scans were not de-identified prior to preprocessing, but were de-identified (i.e., de-faced) using the PyDeface software package (Gulban *et al.*, 2022) prior to sharing data on OpenNeuro (https://openneuro.org/datasets/ds004697/versions/1.0.2).

Anatomical data preprocessing. A total of 1 T1-weighted (T1w) images were found within the input BIDS dataset.The T1-weighted (T1w) image was corrected for intensity non-uniformity (INU) with N4BiasFieldCorrection (Tustison et al. 2010), distributed with ANTs 2.3.3 (Avants et al. 2008, RRID:SCR_004757), and used as T1w-reference throughout the workflow. The T1w-reference was then skull-stripped with a Nipype implementation of the antsBrainExtraction.sh workflow (from ANTs), using OASIS30ANTs as target template. Brain tissue segmentation of cerebrospinal fluid (CSF), white-matter (WM) and gray-matter (GM) was performed on the brain-extracted T1w using fast (FSL 5.0.9, RRID:SCR_002823, Zhang, Brady, and Smith 2001). Brain surfaces were reconstructed using recon-all (FreeSurfer 6.0.1, RRID:SCR_001847, Dale, Fischl, and Sereno 1999), and the brain mask estimated previously was refined with a custom variation of the method to reconcile ANTs-derived and FreeSurfer-derived segmentations of the cortical gray-matter of Mindboggle (RRID:SCR_002438, Klein et al. 2017). Volume-based spatial normalization to three standard spaces (MNIPediatricAsym:cohort-3, MNI152NLin2009cAsym, MNI152NLin6Asym) was performed through nonlinear registration with antsRegistration (ANTs 2.3.3), using brain-extracted versions of both T1w reference and the T1w template. The following templates were selected for spatial normalization: MNI's unbiased standard MRI template for pediatric data from the 4.5 to 18.5y age range [(???), RRID:SCR_008796; TemplateFlow ID: MNIPediatricAsym:cohort-3], ICBM 152 Nonlinear Asymmetrical template version 2009c [Fonov et al. (2009), RRID:SCR_008796; TemplateFlow ID: MNI152NLin2009cAsym], FSL's MNI ICBM 152 non-linear 6th Generation Asymmetric Average Brain Stereotaxic Registration Model [Evans et al. (2012), RRID:SCR_002823; TemplateFlow ID: MNI152NLin6Asym],

Functional data preprocessing. For each of the 6 BOLD runs found per subject (across all tasks and sessions), the following preprocessing was performed. First, a reference volume and its skull-stripped version were generated using a custom methodology of fMRIPrep. A B0-nonuniformity map (or fieldmap) was estimated based on a phase-difference map calculated with a dual-echo GRE (gradient-recall echo) sequence, processed with a custom workflow of SDCFlows inspired by the epidewarp.fsl script and further improvements in HCP Pipelines (Glasser et al. 2013). The fieldmap was then co-registered to the target EPI (echo-planar imaging) reference run and converted to a displacements field map (amenable to registration tools such as ANTs) with FSL's fugue and other SDCflows tools[1]. Based

[1] Fieldmap-based susceptibility distortion correction (SDC) was used for all but one subject (N = 62). For one subject, *fieldmap-less* SDC was used due to a low-quality fieldmap (i.e., motion) and improved fMRIPrep output using the fieldmap-less approach. For this approach, a deformation field to correct for susceptibility distortions was

on the estimated susceptibility distortion, a corrected EPI (echo-planar imaging) reference was calculated for a more accurate co-registration with the anatomical reference. The BOLD reference was then co-registered to the T1w reference using bbregister (FreeSurfer) which implements boundary-based registration (Greve and Fischl 2009). Co-registration was configured with six degrees of freedom. Head-motion parameters with respect to the BOLD reference (transformation matrices, and six corresponding rotation and translation parameters) are estimated before any spatiotemporal filtering using mcflirt (FSL 5.0.9, Jenkinson et al. 2002). BOLD runs were slice-time corrected using 3dTshift from AFNI 20210206 (Cox and Hyde 1997, RRID:SCR_005927). The BOLD time-series were resampled onto the following surfaces (FreeSurfer reconstruction nomenclature): fsaverage5. The BOLD time-series (including slice-timing correction when applied) were resampled onto their original, native space by applying a single, composite transform to correct for head-motion and susceptibility distortions. These resampled BOLD time-series will be referred to as preprocessed BOLD in original space, or just preprocessed BOLD. The BOLD time-series were resampled into several standard spaces, correspondingly generating the following spatially-normalized, preprocessed BOLD runs: MNIPediatricAsym:cohort-3, MNI152NLin2009cAsym. First, a reference volume and its skull-stripped version were generated using a custom methodology of fMRIPrep. Automatic removal of motion artifacts using independent component analysis (ICA-AROMA, Pruim et al. 2015) was performed on the preprocessed BOLD on MNI space time-series after removal of non-steady state volumes and spatial smoothing with an isotropic, Gaussian kernel of 6mm FWHM (full-width half-maximum). Corresponding "non-aggresively" denoised runs were produced after such smoothing. Additionally, the "aggressive" noise-regressors were collected and placed in the corresponding confounds file. Several confounding time-series were calculated based on the preprocessed BOLD: framewise displacement (FD), DVARS and three region-wise global signals. FD was computed using two formulations following Power (absolute sum of relative motions, Power et al. (2014)) and Jenkinson (relative root mean square displacement between affines, Jenkinson et al. (2002)). FD and DVARS are calculated for each functional run, both using their implementations in Nipype (following the definitions by Power et al. 2014). The three global signals are extracted within the CSF, the WM, and the whole-brain masks. Additionally, a set of physiological regressors were extracted to allow for component-based noise correction (CompCor, Behzadi et al. 2007). Principal components are estimated after high-pass filtering the preprocessed BOLD time-series (using a discrete cosine filter with 128s cut-off) for the two CompCor variants: temporal (tCompCor) and anatomical (aCompCor). tCompCor components are then calculated from the top 2% variable voxels within the brain mask. For aCompCor, three probabilistic masks (CSF, WM and combined CSF+WM) are generated in anatomical space. The implementation differs from that of Behzadi et al. in that instead of eroding the masks by 2 pixels on BOLD space, the aCompCor masks are subtracted a mask of pixels that likely contain a volume fraction of GM. This mask is obtained by dilating a GM mask extracted from the FreeSurfer's aseg segmentation, and it ensures components are not extracted from voxels containing a minimal fraction of GM. Finally, these masks are resampled into BOLD space and binarized by thresholding at 0.99 (as in the original implementation). Components are also calculated separately within the WM and CSF masks. For each CompCor decomposition, the k components with the largest singular values are retained, such that the retained components' time series are sufficient to explain 50 percent of variance across the nuisance mask (CSF, WM, combined, or temporal). The remaining components are dropped from consideration. The head-

---

estimated based on *fMRIPrep*'s *fieldmap-less* approach. The deformation field is that resulting from co-registering the BOLD reference to the same-subject T1w-reference with its intensity inverted (Wang et al. 2017; Huntenburg 2014). Registration is performed with antsRegistration (ANTs 2.3.3), and the process regularized by constraining deformation to be nonzero only along the phase-encoding direction, and modulated with an average fieldmap template (Treiber et al. 2016).

motion estimates calculated in the correction step were also placed within the corresponding confounds file. The confound time series derived from head motion estimates and global signals were expanded with the inclusion of temporal derivatives and quadratic terms for each (Satterthwaite et al. 2013). Frames that exceeded a threshold of 0.5 mm FD or 1.5 standardised DVARS were annotated as motion outliers. All resamplings can be performed with a single interpolation step by composing all the pertinent transformations (i.e. head-motion transform matrices, susceptibility distortion correction when available, and co-registrations to anatomical and output spaces). Gridded (volumetric) resamplings were performed using antsApplyTransforms (ANTs), configured with Lanczos interpolation to minimize the smoothing effects of other kernels (Lanczos 1964). Non-gridded (surface) resamplings were performed using mri_vol2surf (FreeSurfer).

Many internal operations of fMRIPrep use Nilearn 0.6.2 (Abraham et al. 2014, RRID:SCR_001362), mostly within the functional processing workflow. For more details of the pipeline, see the section corresponding to workflows in fMRIPrep's documentation.

Laboratory portion size meals

Table S1. Amount of food served by portion size condition

	Reference portion	33% increase from reference portion	66% increase from reference portion	99% increase from reference portion
Total amount served, grams	769	1011.4	1255.8	1492.2
Total amount served, kcal	1048	1376.8	1706.6	2030.4

Analyses

Imputation of missing pre-MRI fullness value. For one subject with missing data, **t**hree pre-MRI fullness values were imputed from sex, age, and BMI percentile using multivariate imputation by chained equations in R (Buuren and Groothuis-Oudshoorn, 2011) with 5 iterations per imputation. As group-level responses to portion size were not impacted by the imputed values (Fuchs *et al.*, under review), the median value was used in the present analyses.

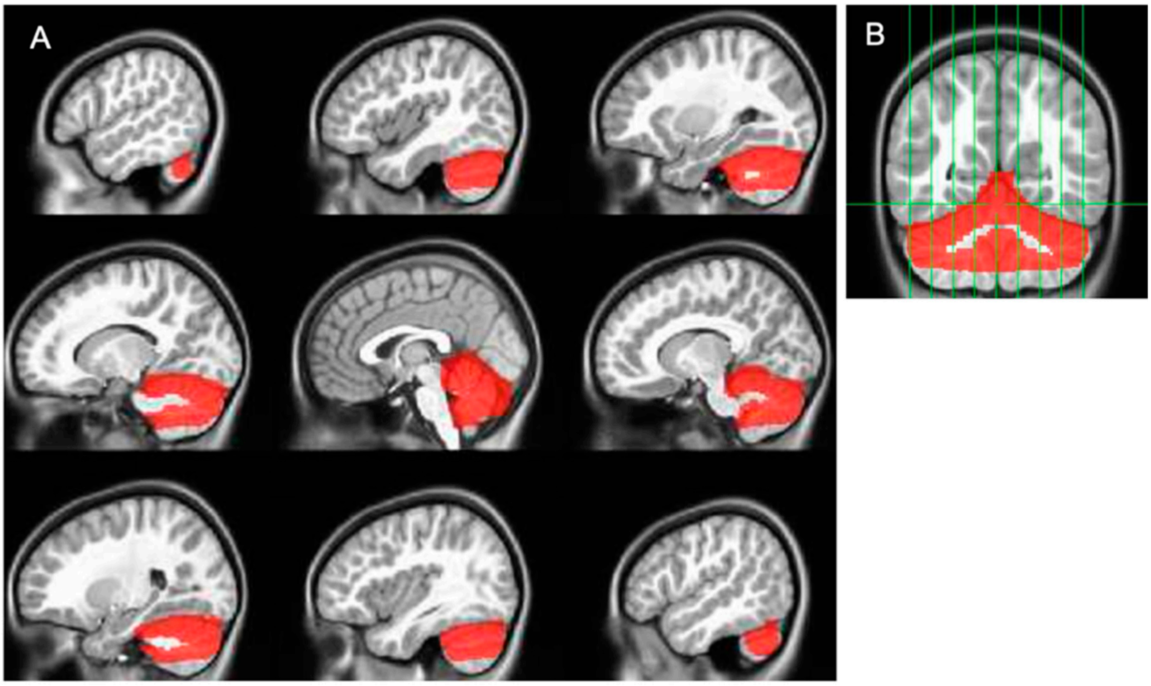

Figure S1. Cerebellum mask: (a) Sagittal slices with cerebellum mask (red) overlayed on MNI's unbiased template for pediatric cohort 3. Regions included in mask are listed in Table 1; (b) Coronal slice with cerebellum mask (red) overlayed on MNI's unbiased template for pediatric cohort 3. Green vertical lines indicate location of sagittal slices depicted in (a)

Results

Descriptive Statistics

Table S2. Pearson correlation coefficients between individual-level portion size slopes

	1	*2*	*3*	*4*
Linear portion size slope, estimated from g	-			
Linear portion size slope, estimated from kcal	0.76***	-		
Quadratic portion size slope, estimated from g	0.01	-0.2	-	
Quadratic portion size slope, estimated from kcal	-0.02	-0.22	0.79***	-

** FDR-adjusted p < 0.05, **FDR- adjusted p < 0.01, *** FDR-adjusted p < 0.001.*

Table S3. Associations between portion size slopes and child characteristics

	Linear slope, g (n = 61)	Linear slope, kcal (n = 61)	Quadratic slope, g (n = 58)	Quadratic slope, kcal (n = 58)
Age, r [a]	0.07	0.02	0.21	-0.07
BMI percentile, r [a]	-0.12	0.02	-0.15	-0.17
Sex, t-stat (df) [b]	-0.92 (60.7)	0.32 (60.7)	-1.00 (55.9)	0.60 (58.0)

All p-values were > 0.1 (unadjusted)
[a] Associations assessed with Pearson correlations
[b] Associations assessed with 2-sample t-tests (equal variance between groups not assumed); male participants were treated as the reference group

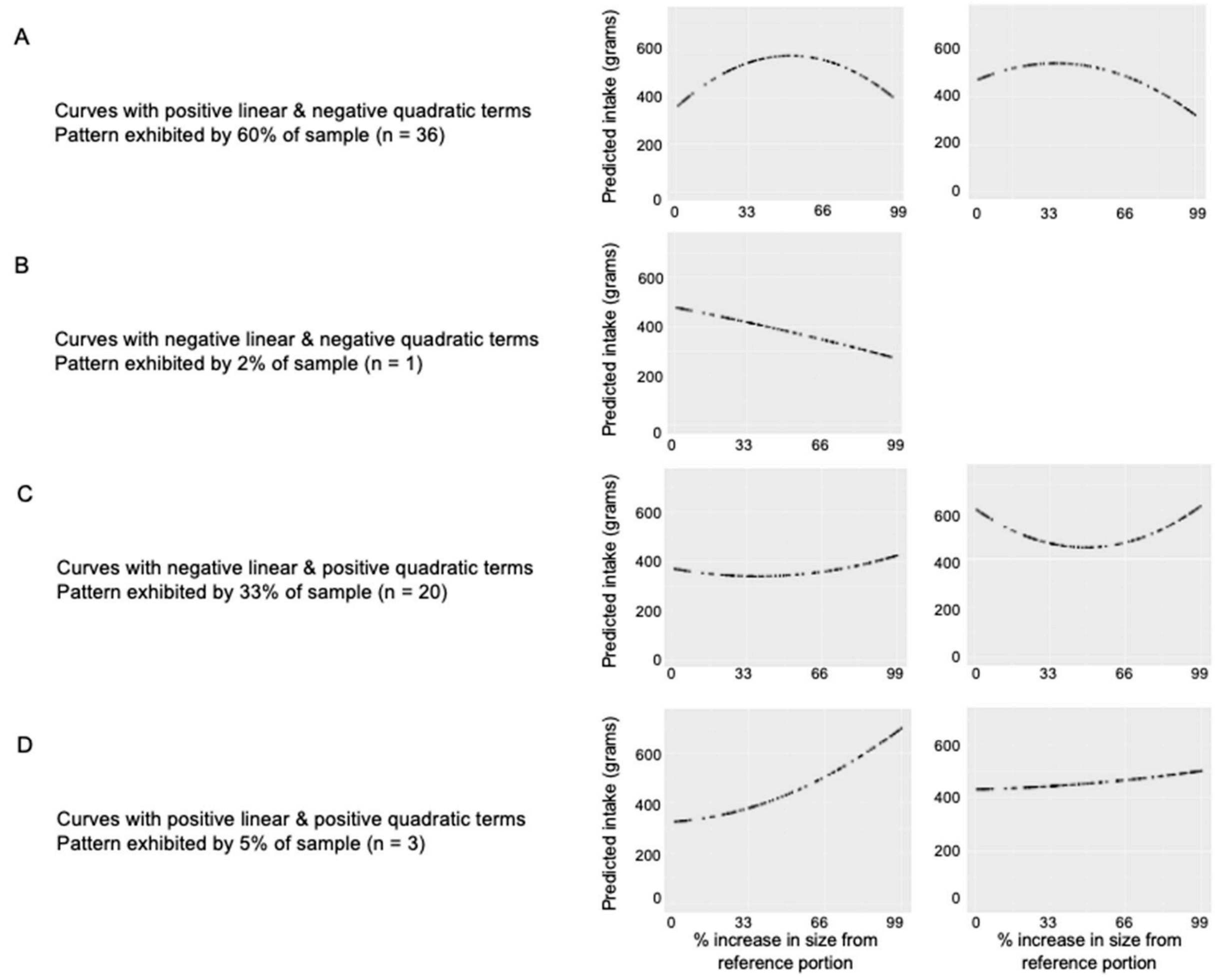

Figure S2. Example portion size curves for gram intake. Graphs show predicted intake in grams (y-axis) by % increase in portion size relative to the study's reference condition (x-axis). Data for each curve was simulated using individual-level intercepts and slopes derived from fixed-effects individual slopes (FEIS) models predicting intake (grams) from the linear and quadratic increases in portion size from the reference portion [0, 33%, 66%, 99%], controlling for pre-meal fullness, average liking of foods at the meal, and meal order. Graphs show examples of curves characterized by (A) positive linear and negative quadratic terms, (B) negative linear and negative quadratic terms, (C) negative linear and positive quadratic terms, and (D) positive linear and positive quadratic terms.

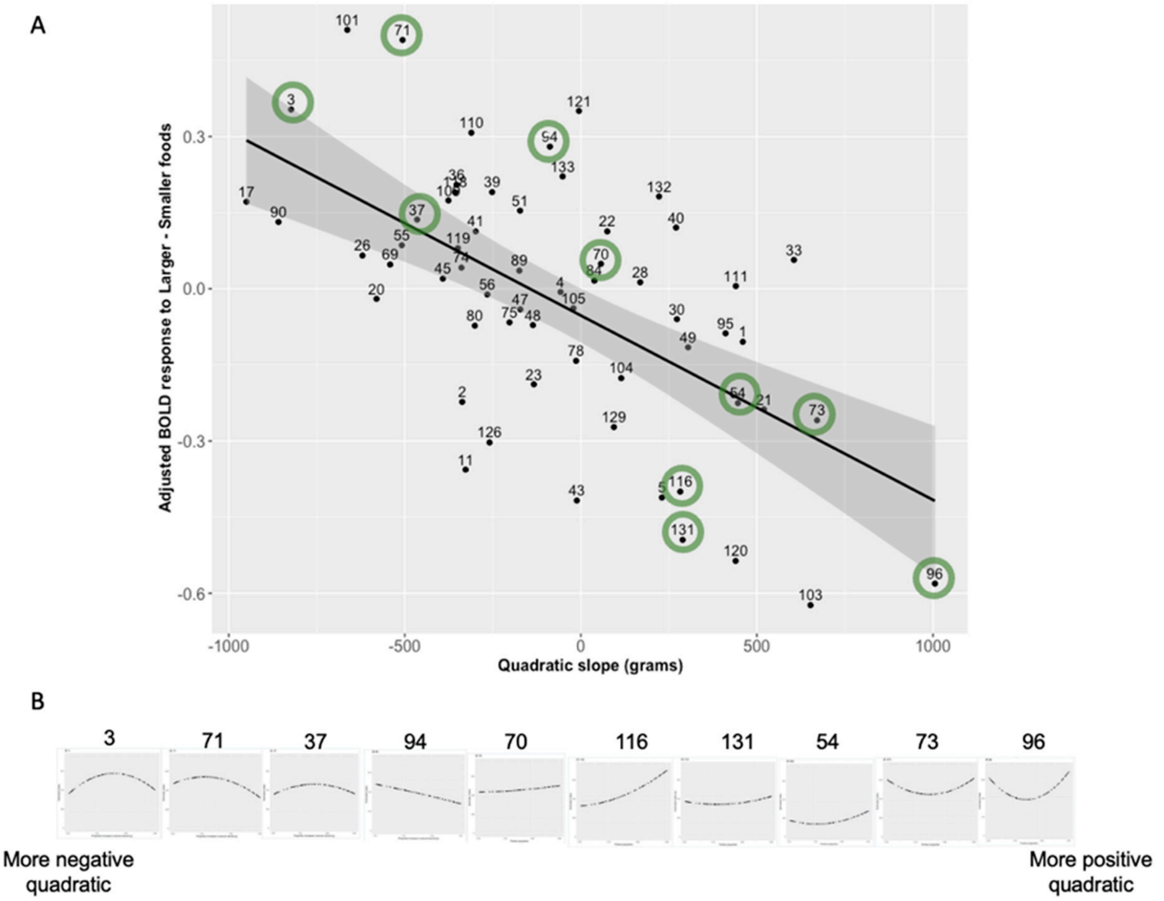

Figure S3. Association between cerebellar response to portion size and quadratic portion size slope with example portion size curves. (A) Scatterplot showing association between cerebellar response to portion size (larger – smaller) and quadratic portion size slope; x-axis: individual-level quadratic portion size slope extracted from a fixed-effects individual slopes (FEIS) model predicting intake (grams) from the linear and quadratic increases in portion size from the reference portion [0, 33%, 66%, 99%], controlling for pre-meal fullness, average liking of foods at the meal, and meal order; y-axis: cerebellar BOLD response to portion size (larger – smaller; y-axis) adjusted for sex, average framewise displacement, pre-MRI fullness, pre-MRI anxiety and intercept and linear slope parameters from the quadratic FEIS model. This is the same association shown in Figure 2 (main text), but with datapoints circled for 10 children (circled in green). (B). Portion size curves plotted for the 10 children circled in (A). Data for each curve was simulated using individual-level intercepts and slopes derived from the quadratic FEIS model. Subject IDs above each point in (A) correspond to the ID above the portion size curves in (B).

Citations

Buuren, S. van, Groothuis-Oudshoorn, K. (2011). mice: Multivariate Imputation by Chained Equations in R. *Journal of Statistical Software*, **45**, 1–67

Fuchs, B.A., Pearce, A.L., Rolls, B.J., et al. (under review). Does ‘portion size’ matter? Neural responses to food and non-food cues presented in varying amounts. *Appetite*

Gulban, O.F., Nielson, D., lee, john, et al. (2022). poldracklab/pydeface: PyDeface v2.0.2
